# Potent Antioxidant and Genoprotective Effects of Boeravinone G, a Rotenoid Isolated from *Boerhaavia diffusa*

**DOI:** 10.1371/journal.pone.0019628

**Published:** 2011-05-20

**Authors:** Gabriella Aviello, Jasna M. Canadanovic-Brunet, Natasa Milic, Raffaele Capasso, Ernesto Fattorusso, Orazio Taglialatela-Scafati, Ines Fasolino, Angelo A. Izzo, Francesca Borrelli

**Affiliations:** 1 Department of Experimental Pharmacology, University of Naples Federico II, Naples, Italy; 2 Faculty of Technology, University of Novi Sad, Novi Sad, Serbia; 3 Department of Natural Products Chemistry, University of Naples Federico II, Naples, Italy; Enzo Life Sciences, Inc., United States of America

## Abstract

**Background and Aims:**

Free radicals are implicated in the aetiology of some gastrointestinal disorders such as gastric ulcer, colorectal cancer and inflammatory bowel disease. In the present study we investigated the antioxidant and genoprotective activity of some rotenoids (i.e. boeravinones) isolated from the roots of *Boerhaavia diffusa*, a plant used in the Ayurvedic medicine for the treatment of diseases affecting the gastrointestinal tract.

**Methods/Principal Findings:**

Antioxidant activity has been evaluated using both chemical (Electron Spin Resonance spectroscopy, ESR) and Caco-2 cells-based (TBARS and ROS) assays. DNA damage was evaluated by Comet assay, while pERK_1/2_ and phospho-NF-kB p65 levels were estimated by western blot. Boeravinones G, D and H significantly reduced the signal intensity of ESR induced by hydroxyl radicals, suggesting a scavenging activity. Among rotenoids tested, boeravinone G exerted the most potent effect. Boeravinone G inhibited both TBARS and ROS formation induced by Fenton's reagent, increased SOD activity and reduced H_2_O_2_-induced DNA damage. Finally, boeravinone G reduced the levels of pERK_1_ and phospho-NF-kB p65 (but not of pERK_2_) increased by Fenton's reagent.

**Conclusions:**

It is concluded that boeravinone G exhibits an extraordinary potent antioxidant activity (significant effect in the nanomolar range). The MAP kinase and NF-kB pathways seem to be involved in the antioxidant effect of boeravinone G. Boeravinone G might be considered as lead compound for the development of drugs potentially useful against those pathologies whose aetiology is related to ROS-mediated injuries.

## Introduction

Free radicals are (usually) highly reactive atomic or molecular species with an unpaired number of electrons. Free radicals have been implicated in the aetiology of various human diseases, including gastrointestinal disorders (such as gastric ulcer, colorectal cancer, inflammatory bowel disease, etc.) [1]–[5] and play an important role as mediators of inflammation [6]. The gastrointestinal tract is particularly well endowed with the enzymatic machinery necessary to form large amounts of oxygen radicals, *e.g.* mucosal xanthine oxidase and NADPH oxidase, which occur in resident phagocytotic leukocytes of the *lamina propria*. A number of studies have shown that certain indices of oxidative stress (e.g. malondialdehyde, phospholipase A2 and myeloperoxidase) are increased in intestinal tissues of patients with disorders of the digestive system [7]–[10]. Other studies have reported a relationship between glutathione depletion and gut diseases [11]–[13]. Therefore, a new approach for the treatment of gastrointestinal diseases could involve the use of antioxidants (to prevent and neutralise toxic oxygen intermediates) or drugs which increase the functionality of endogenous antioxidant systems (such as superoxide dismutase, catalase or glutathione peroxidase).

*Boerhaavia diffusa* is a herbaceous member of the Nyctaginaceae family which has a long history of use by indigenous and tribal people of Brazil and India [14]. In particular, roots and leaves of this plant have been widely used in the folk medicine to treat several illnesses including those affecting the gastrointestinal tract (dyspepsia, abdominal pain, etc.). Experimental studies have demonstrated that *B. diffusa* could be effective in the prevention and treatment of diseases in which oxidants or free radicals are implicated (i.e. inflammation, cancer, diabetes, etc.) [15]–[19]. The main chemical ingredients of this plant include alkaloids (punarnavine), rotenoids (boeravinones A to J) and flavones [20]. In light of these data, in this paper we evaluated the antioxidant/genoprotective effect of *B. diffusa* and tried to identify the active ingredients responsible of its activity.

## Results

### Preparation of the methanol extract and isolation of rotenoids

**Figure 1:**
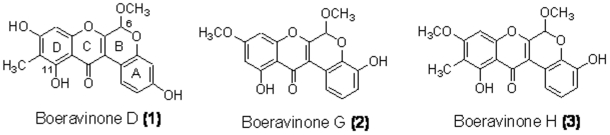
Chemical structure of the most potent antioxidant rotenoids. Rotenoids were obtained from Kupchan partitioning of the methanol extract of *B. diffusa* root following by sequential silica gel column chromatography and HPLC.

Roots of *Boerhaavia diffusa* (2.20 lbs) were extracted with methanol (3×3 L) at room temperature and the obtained extract was subjected to Kupchan partitioning to obtain four different fractions (*n*-hexane, CCl_4_, CHCl_3_, *n*-BuOH). Preliminary antioxidant assays showed that the CCl_4_ fraction possessed high antioxidant activity, therefore, this fraction was further separated. ESR-guided (see below) purification through sequential silica gel column chromatography and HPLC as detailed in the Experimental Section led to the isolation of boeravinone D (**1**, 6.4 mg), boeravinone G (**2**, 7.5 mg) and boeravinone H (**3**, 5.2 mg) (Fig. 1). The structures of these molecules were identified on the basis of the comparison of their spectral data with those reported in the literature [21], [22].

### Electron spin resonance spectroscopy

**Figure 2:**
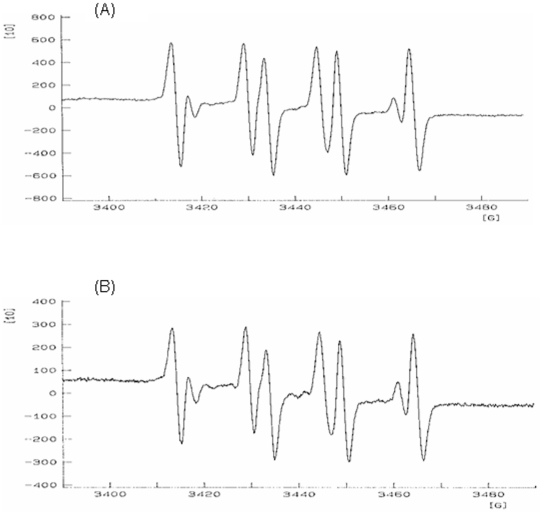
Effect of 5 mg/ml of *Boerhaavia diffusa* methanol extract on *electron spin resonance spectroscopy*. Representative ESR spectra of DMPO-OH spin adduct signal (A) and DMPO-OH spin adduct signal in the presence of 5 mg/ml of *Boerhaavia diffusa* methanol extract (B).

As shown in Figure 2, the reaction of Fe^2+^ and H_2_O_2_ in the presence of the spin trapping agent DMPO, generated a 1∶2∶2∶1 quartet of lines in the ESR spectrum with the hyperfine coupling parameters (a_N_ and a_H_ = 14.9 G). BDME (0.1–5 mg/ml) produced a concentration-dependent inhibition of the ESR signal intensity of DMPO-OH spin adduct (antioxidant activity (AA) %: BDME 0.1 mg/ml 12.2±0.51, BDME 0.5 mg/ml 26.55±0.62, BDME 1 mg/ml 52.32±0.98, BDME 3 mg/ml 66.9±0.57, BDME 5 mg/ml 71.22±0.43, n = 3) (Fig. 2). Among the four fractions (*n*-hexane, CCl_4_, CHCl_3_, *n*-BuOH) obtained from BDME through a modified Kupchan partitioning procedure (see Materials and Methods), the CCl_4_ and the *n*-BuOH ones were able to reduce the ESR signal intensity, with the former more active (Table 1). The carbon tetrachloride extract produced a total elimination of hydroxyl radical (AA = 100%) at the concentration of 0.7 mg/ml. Chromatographic purification of the CCl_4_ fraction through column chromatography on silica gel (see Materials and Methods) led to the formation of 13 sub-fractions whose antioxidant activity was evaluated through ESR assay (Table 2). HPLC purification of the most active sub-fractions (BOE4, BOE6, BOE 8 and BOE 9) led to the isolation of boeravinone G, boeravinone H and boeravinone D (structures in Fig. 1) which, at the concentration of 0.5 mg/ml, showed a scavenger activity of 65.9±3.3%, 50.2±2.4% and 48.6±1.4%, respectively.

**Table 1:** Antioxidant activity (AA), detected using ESR assay, of the fractions obtained from Kupchan partitioning of the methanol extract of *B. diffusa* root.

Extracts	AA (%)				
	0.1 mg ml^−1^	0.5 mg ml^−1^	1 mg ml^−1^	3 mg ml^−1^	5 mg ml^−1^
n-Hexane	0	0	0	0	0
Chloroform	0	0	0	0	0
Carbon tetrachloride	59.87±0.65	85.45±2.26	100±0	100±0	100±0
*n*-Butanol	15.50±0,39	35.56±0.88	61.22±1.66	68.90±1.85	78.1±1.69

**Table 2:** Antioxidant activity (AA), detected using ESR assay, of the fractions obtained from the carbon tetrachloride extract of *B. diffusa* root.

CCl_4_ fractions	AA (%)		
	0.01 mg ml^−1^	0.15 mg ml^−1^	1 mg ml^−1^
BOE 1	26.32±0.44	76.87±1.28	91.50±1.52
BOE 2	24.41±0.41	78.05±1.30	92.20±1.55
BOE 3	23.22±0.39	77.45±1.29	88.45±1.47
BOE 4	31.24±0.52	82.93±1.39	98.75±0.48
BOE 5	20.13±0.33	65.85±1.09	82.31±1.37
BOE 6	30.12±0.50	81.71±1.36	100±0
BOE 7	24.13±0.0.40	75.61±1.26	86.71±1.44
BOE 8	33.34±0.55	82.32±1.37	100±0
BOE 9	32.98±0.54	81.71±1.36	100±0
BOE 10	0	12.19±0.20	51.25±0.85
BOE 11	22.32±0.37	75.61±1.26	90.81±1.51
BOE 12	15.12±0.25	51.22±0.85	75.25±1.25
BOE 13	20.12±0.33	75.61±1.26	88.45±1.48

### Lipid peroxidation

**Figure 3:**
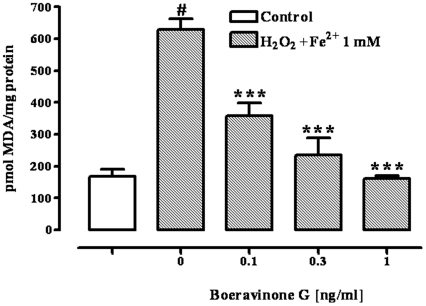
Effect of boeravinone G (0.1–1 ng/ml) on Fenton's reagent (H_2_O_2_/Fe^2+^ 1 mM)-induced malondialdehyde-equivalents (MDA-equivalents) production. Effect observed in differentiated Caco-2 cells after 24-hour boeravinone G exposure. Data represent mean ± SEM of 6 experiments. ^#^p<0.001 *vs* control (vehicle) and ***p<0.001 *vs* H_2_O_2_/Fe^2+^ alone.

The treatment with H_2_O_2_/Fe^2+^ (1 mM) produced a significant (p<0.001) threefold increase in TBARS formation (Fig. 3). Boeravinone G (0.1–1 ng/ml) significantly and in a concentration-related manner (p<0.001) reduced H_2_O_2_/Fe^2+^-induced TBARS formation (Fig. 3). Boeravinone G given alone (i.e. in absence of H_2_O_2_/Fe^2+^ treatment), at all concentrations used, did not modify the TBARS levels (pmol MDA/mg protein: control 168.5±21.35, boeravinone G 0.1 ng/ml 175.8±13.54, boeravinone G 0.3 ng/ml 173.3±17.21, boeravinone G 1 ng/ml 171.8±14.16; n = 6).

### Intracellular ROS

**Figure 4:**
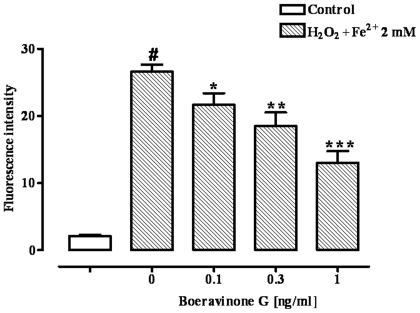
Effect of boeravinone G (0.1–1 ng/ml) on Fenton's reagent (H_2_O_2_/Fe^2+^ 2 mM)-induced reactive species (ROS) production. Effect observed in differentiated Caco-2 cells after 24-hour boeravinone G exposure. Data represent mean ± SEM of 6 experiments. ^#^p<0.001 *vs* control (vehicle); *p<0.05, **p<0.01 and ***p<0.001 *vs* H_2_O_2_/Fe^2+^ alone.

Exposure of Caco-2 cells to H_2_O_2_/Fe^2+^ (2 mM) produced a significant (p<0.001) increase in ROS formation (Fig. 4). A pre-treatment for 24 h with boeravinone G (0.1–1 ng/ml) reduced the ROS formation significantly (p<0.05-0.001) and in a concentration dependent manner as measured by the inhibition of DCF fluorescence intensity (Fig. 4). Boeravinone G (0.1–1 ng/ml), given alone (i.e. in absence of H_2_O_2_/Fe^2+^ treatment), did not affect the formation of ROS (Fluorescence intensity: control 2.45±0.09, boeravinone G 0.1 ng/ml 2.45±0.14, boeravinone G 0.3 ng/ml 2.36±0.17, boeravinone G 1 ng/ml 2.42±0.09; n = 6).

### DNA damage

**Figure 5:**
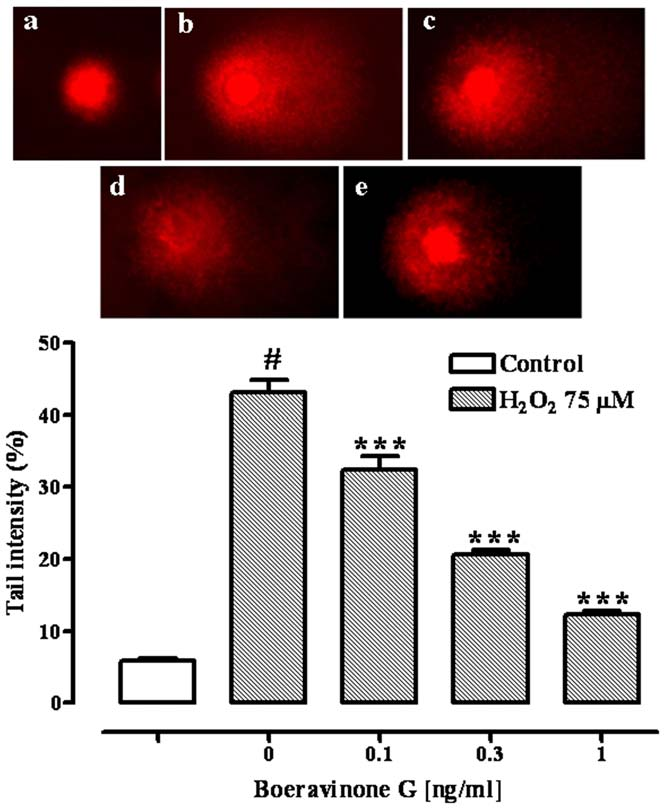
Effect of boeravinone G (BG, 0.1–1 ng/ml) on DNA damage. DNA damage (tail intensity) was detected by the Comet assay in Caco-2 cells exposed to 75 µM H_2_O_2_ for 5 min in absence or presence of boeravinone G. a = control; b = H_2_O_2_ 75 µM; c = H_2_O_2_ 75 µM+BG 0.1 ng/ml; d = H_2_O_2_ 75 µM+BG 0.3 ng/ml; e = H_2_O_2_ 75 µM+BG 1 ng/ml. Data represent mean ± SEM of 4 experiments. ^#^p<0.001 *vs* control (vehicle) and ***p<0.001 *vs* H_2_O_2_ alone.

Boeravinone G (0.1–1 ng/ml) did not produce DNA damage detected by the Comet assay in Caco-2 cells (% tail intensity: control 5.37±0.26, boeravinone G 0.1 ng/ml 5.29±0.19, boeravinone G 0.3 ng/ml 5.21±0.22, boeravinone G 1 ng/ml 5.32±0.25; n = 4), excluding a genotoxic effect. Exposure of the Caco-2 cells to H_2_O_2_ (75 µM) produced a significant (p<0.001) DNA damage (Fig. 5), expressed as comet tail intensity. Tail DNA fluorescence of H_2_O_2_-damaged Caco-2 cells was about 43%, while the control was about 5%. A pre-treatment with boeravinone G (0.1–1 ng/ml) reduced significantly (p<0.001) and in a concentration dependent manner the DNA damage induced by H_2_O_2_ (Fig. 5). Consistent with the TBARS assay, a significant inhibitory effect was achieved for the 0.1–1 ng/ml (boeravinone G) concentrations.

### SOD activity

**Figure 6:**
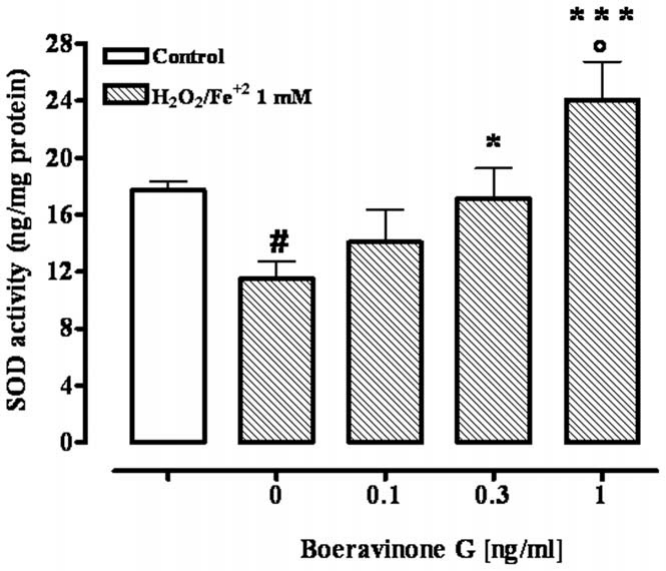
Effect of boeravinone G (0.1–1 ng/ml) on superoxide dismutase (SOD) activity. SOD activity was evaluated in Caco-2 cells exposed to Fenton's reagent (H_2_O_2_/Fe^2+^ 1 mM) without or with boeravinone G (0.1–1 ng/ml). Data represent mean ± SEM of 4 experiments. ^#^p<0.001 *vs* control (vehicle); *p<0.05 and ***p<0.001 *vs* H_2_O_2_/Fe^2+^ alone; °p<0.05 *vs* control.

Twenty-four hours exposure of Caco-2 cells to H_2_O_2_/Fe^2+^ (1 mM) produced a significant (p<0.001) decrease in SOD activity which was concentration-dependently counteracted by boeravinone G (Fig. 6). Interestingly, boeravinone G, at the highest concentration tested (1 ng/ml), resulted in a SOD activity which was significantly higher than the value measured in untreated cells (i.e. cells not treated with H_2_O_2_/Fe^2+^) (Fig. 6). Boeravinone G (0.1–1 ng/ml), used alone (i.e. in absence of H_2_O_2_/Fe^2+^ treatment), did not modify the activity of SOD [SOD activity (ng/mg protein): control 17.7±0.64, boeravinone G 0.1 ng/ml 18.02±0.90, boeravinone G 0.3 ng/ml 17.86±0.96, boeravinone G 1 ng/ml 17.2±0.79; n = 6].

### pERK_1/2_ and phospho-NF-kB p65 expressions

**Figure 7:**
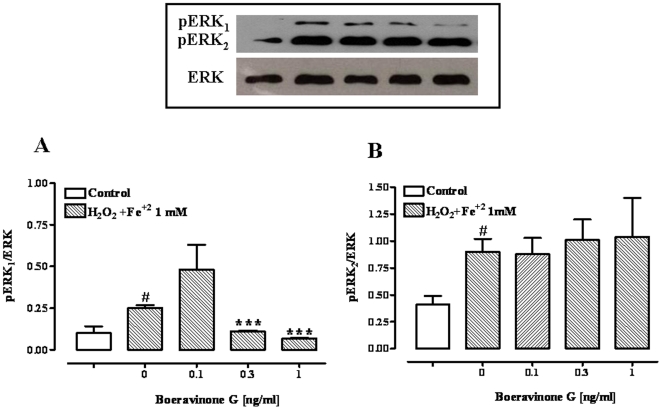
Effect of boeravinone G (0.1–1 ng/ml) on pERK_1_ (A) and pERK_2_ (B) expression. Quantitative analysis and representative western blot analysis of pERK_1_ and pERK_2_ in Caco-2 cells exposed to Fenton's reagent (H_2_O_2_/Fe^2+^ 1 mM) without or with boeravinone G (0.1–1 ng/ml). The results were normalized with anti-ERK_2_ (pERK_1/2_/ERK_2_). ^#^p<0.01 *vs* control (vehicle); ***p<0.001 *vs* H_2_O_2_/Fe^2+^ alone.

**Figure 8:**
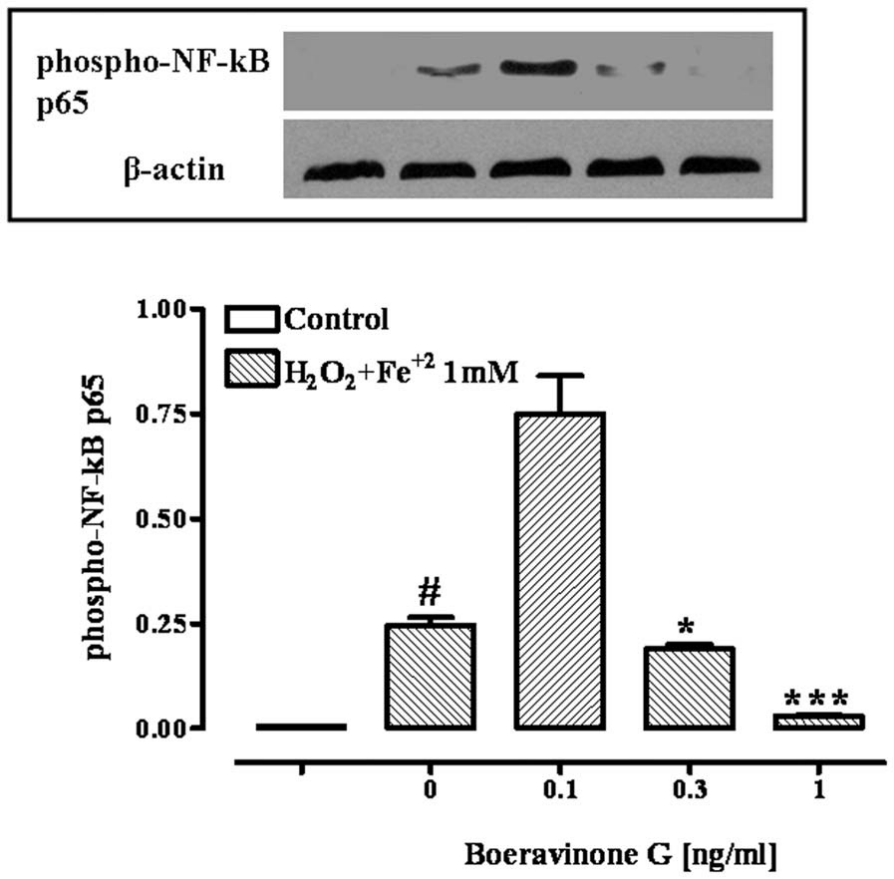
Effect of boeravinone G (0.1–1 ng/ml) on phospho-NF-kB p65 expression. Quantitative analysis and representative western blot analysis of phospho-NF-kB p65 in Caco-2 cells exposed to Fenton's reagent (H_2_O_2_/Fe^2+^ 1 mM) without or with boeravinone G (0.1–1 ng/ml). The results were normalized with anti-βactin antibodies. ^#^p<0.001 *vs* control (vehicle); *p<0.05 and ***p<0.001 *vs* H_2_O_2_/Fe^2+^ alone.

Fenton's reagent (H_2_O_2_/Fe^2+^ 1 mM) elicited a significant increase in the levels of phosphorylated ERK_1_ (pERK_1_), ERK_2_ (pERK_2_) and NF-kB p65 (Figure 7 and 8). Boeravinone G (at 0.3 and 1 ng/ml) significantly reduced the levels of pERK_1_ and phospho-NF-kB p65 (Figure 7 and 8). However, at the lower concentration of boheravinone G tested (i.e., 0.1 ng/ml), increased levels of both pERK1 and phospho-NF-kB p65 were observed (Figure 7 and 8). By contrast, boeravinone G, at all the concentration evaluated (0.1–1 ng/ml), did not affect H_2_O_2_/Fe^2+^ -induced pERK_2_ up-regulation (Figure 7).

### Cytotoxicity assays

The exposure of Caco-2 cells to various concentrations of the most active antioxidant rotenoid boeravinone G (0.1–1 ng/ml) resulted in no effect on cell survival (% cell survival: control 100±0, boeravinone G 0.1 ng/ml 97.2±4.21, boeravinone G 0.3 ng/ml 100.3±2.62, boeravinone G 1 ng/ml 99.7±3.69, n = 6).

Boeravinone G (0.1–1 ng/ml) did not produce any increase in the release of LDH from Caco-2 cell line (% LDH leakage: control 11.5±0.51, boeravinone G 0.1 ng/ml 9.8±0.39, boeravinone G 0.3 ng/ml 10.4±0.46, boeravinone G 1 ng/ml 10.8±0.55, n = 6).

## Discussion

Gastrointestinal diseases (such as gastric ulcer, colorectal cancer and inflammatory bowel disease) are important public health problems all over the world. There is a growing evidence that oxygen-derived free radicals play an important role in the pathogenesis of the digestive system disorders. Studies investigating the role of ROS in patients with IBD, have reported the presence of abnormal high levels of these radicals [23], [24]. Consistently, antioxidant compounds (*e.g.* polyphenols from green tea) are believed to prevent a number of gastrointestinal diseases [25]–[28] and drugs with antioxidant activity are currently used in the treatment of these diseases [29]–[31].

In the present study, we have found that *Boerhaavia diffusa*, an Ayurvedic herbal medicine used for the treatment of several gastrointestinal diseases, possesses remarkable antioxidant and genoprotective properties which could contribute to explain, at least in part, its traditional use in gastrointestinal ailments; moreover, for the first time, we have demonstrated that rotenoids, mainly boeravinone G, are responsible of this activity.

One of the approaches to assess the antioxidant activity is to examine directly free radical production and inhibition by using the highly sensitive electron spin resonance (ESR) spectroscopy, which is able to detect the presence and concentration of oxygen free radicals directly. Since hydroxyl radicals are very unstable, an exogenous spin trap reacting with the free radical species was used, thus generating more stable adducts with characteristic ESR profiles. In the present study, it was found that a methanol extract of *B. diffusa* (BDME) reduced the signal intensity of ESR, thus suggesting a scavenging activity. Crude extracts obtained from different parts of the plant (i.e. leaves) have already been shown to exert antioxidant activity in liver and kidney of alloxan-induced diabetic rats and in the liver damaged by acetaminophen [32], [33].

In order to disclose the chemical components of BDME responsible for the antioxidant activity, we have partitioned the methanol extract of *B. diffusa* roots to obtain four fractions (namely *n*-hexane, CCl_4_, CHCl_3_, *n*-BuOH). An ESR-guided fractionation of the most potent antioxidant fraction (CCl_4_) led to the isolation of three rotenoids, boeravinone D [21], G [22] and H [22] with a remarkable radical-scavenging activity. The chemical structures of these compounds are reported in Figure 1. Since our previous investigations revealed that the rotenoid mixture of *B. diffusa* roots is actually made up by at least fifteen compounds [34], [35], we were surprised to notice that only boeravinones D, G, and H seemed to play a major role in the antioxidant activity of the extract. Common features of compounds **1**–**3** are a planar ring C, the presence of free hydroxyl groups on ring A and at position 11, and the presence of a methoxy group at position 6. Remarkably, in the pool of rotenoids present in *B. diffusa* roots [34], [35], boeravinones D, G, and H are the only ones to possess, at the same time, all these features. Moreover, the higher activity of boeravinone G compared to H and D could be ascribed to the absence of the methyl group at position 10 (see figure 1). Since boeravinone G exhibited a higher activity compared to boeravinones D and H, further experiments were performed on this plant compound.

Using the Caco-2 cell line (a human cell line which mimics, after differentiation, the intestinal epithelium) and H_2_O_2_ as a free radical generator, we further investigated the antioxidant effect of boeravinone G by evaluating the lipid peroxidation, assessed as MDA-equivalents, and the production of ROS.

Lipid peroxidation is a complex process that occurs in biological membranes that contain oxidation-susceptible polyunsaturated fatty acids, and leads to the production of lipid hydroperoxides and their metabolites. The cytosolic levels of malondialdehyde and its reactive equivalents are adequate indicators of lipid peroxidation. In the present study, we not only report for the first time the antioxidant activity of boeravinone G in the intestinal cells using the TBARS assay, but also confirm the antioxidant activity using a more specific assay. Specifically, through a fluorescent approach, we have demonstrated that boeravinone G reduced the ROS formation generated by Fenton's reagent. Indeed, although sensitive, the TBARS assay is not specific since many other biological species can react with thiobarbituric acid [35]. Importantly, the antioxidant activity of boeravinone G occurs at nanomolar concentrations, while other well known antioxidant compounds, such as vitamins C and E exert antioxidant activity in the micromolar range [36], [37].

A great number of *in vitro* experiments showed that ROS damages DNA, which appears to represent the major target involved in mutagenesis, carcinogenesis and aging cell responses [38], [39]. Therefore, we also evaluated the potential genoprotective effect of boeravinone G on ROS-induced DNA damage. DNA damage, induced by using H_2_O_2_ (a well-known genotoxic agent able to induce oxidative DNA damage) was evaluated by the Comet assay, which is a very sensitive method for the evaluation of genotoxic/genoprotective effects [40]. Even if we used different concentrations of H_2_O_2_ in the various assays, our experiments suggest that the protective action of boeravinone G, assessed by the TBARS and the ROS assays (see above), could be related to reduction of DNA damage induced by H_2_O_2_. Indeed, boeravinone G was able to reduce H_2_O_2_-induced DNA damage significantly at the concentration of 0.1–1 ng/ml.

In order to investigate the potential targets involved in the boeravinone G antioxidant/genoprotective action, we have analyzed the effect of this plant ingredient on an antioxidant defence enzyme (SOD) and on two signal transduction pathways (MAP kinase and NF-kB) that play a pivotal role in the oxidative stress-induced gastrointestinal disorders [41], [42].

SOD is one of the most effective intracellular enzymatic antioxidants and it acts catalyzing the dismutation of superoxide into oxygen and hydrogen peroxide. According to previous work [43], [44], we have shown a significant decrease in SOD activity in intestinal epithelial cells treated with H_2_O_2_/Fe^2+^. Boeravinone G counteracted the decreased SOD activity thus suggesting a stimulatory effect of this compound on the defence mechanisms of the cells.

When generation of ROS exceeds the capability of the cellular defence systems, several signalling protein kinases and transcription regulatory factors are activated [41], [42], [45]. Indeed, oxidative stress leads to activation of extracellular-signal-related kinases (ERKs) [46]–[48], which are members of the mitogen-activated protein kinase (MAPK) family, and nuclear factor kB (NF-kB) [49]. NF-kB and MAPK are distinct signalling transduction pathways, although, recently, in several situations including oxidative stress, it has been demonstrated a considerable cross talk between these two pathways [50], [51]. We have observed that exposure of Caco-2 to Fenton's reagent leads to an activation of ERK_1_ and ERK_2_. More importantly, we have shown that boeravinone G, at the concentrations of 0.3 and 1 ng/ml, counteracted the increased ERK phosphorylation induced by H_2_O_2_/Fe^2+^-exposure. Surprisingly, the effect of boeravinone G on the ERK phosphorilation was significant only for the 44-kDa isoform pERK_1_ (and not for the pERK_2_ isoform) suggesting a selectivity of action. A differential role for the two kinases in cell signalling has been previously documented [52]. The down-regulation in ERK phosphorylation after boeravinone G exposure is consistent with the observed effect of this compound on SOD activity. Indeed, it is well known the strict correlation existing between Cu-Zn SOD enhancement and ERKs phosphorilation inhibition [53]. Further studies are needed to established if boeravinone G selectively counteracts ROS-mediated ERK and NF-kB activation or, alternatively, if boeravinone G affects the activation of ERK and NF-kB induced by other stimuli (for example, EGFR/RTK-mediated activation).

Similarly, we have here found an increase in phosphorylated NF-kB p65 levels in differentiated Caco-2 cells during the oxidative stress and such increase was counteracted by boeravinone G. The inhibitory effect of boeravinone G on Fenton's reagent-induced phosphorylated p65 up-regulation suggests an involvement of this pathway in the boeravinone G antioxidant activity.

Since boeravinones belong to the chemical class of rotenoids, widely used as botanical insecticides and generally characterized by high toxicity [54], we carried out additional experiments to ensure that boeravinone G, at the concentrations used in our experiments, did not exert any toxic effects. Cytotoxicity was assessed quantitatively by both MTT and LDH assays. We observed no decrease in the cell viability and no increase of LDH release when Caco-2 cells were incubated in the presence of boeravinone G. Moreover, the lack of boeravinone G toxicity has also been demonstrated by the Comet assay since the rotenoid, administered alone (i.e. in absence of damage induced by H_2_O_2_) did not affect DNA integrity. Collectively, these results suggest that boeravinone G was neither cytotoxic nor genotoxic in Caco-2 cells. Accordingly, an interesting study aimed at establishing the “toxophore” of rotenoid molecules, revealed that a prenyl-derived ring attached at ring D and a dimethoxy substitution on ring A are essential requirements [55]. Luckily, both these features are missing in *B. diffusa* rotenoids.

In conclusion, the results obtained in this study demonstrate that BDME exerts antioxidant activity; boeravinone G, H and D appear to be the major compounds responsible for the antioxidant activity, with boeravinone G playing a major role. The genoprotective effect of boeravinone G was associated to a reduction of Fenton's reagent-induced up-regulation of pERK_1_ and NF-kB levels.

In the light of the importance of antioxidant/genoprotective activity in the treatment or prevention of gut disorders and since our experiments were performed on isolated intestinal cells, it is suggested that the antioxidant activity here reported could explain, at least in part, the traditional use of this Ayurvedic remedy in treatment of gastrointestinal disorders (including inflammatory bowel disease and colorectal cancer). Obviously, *in vivo* studies, using well-established animal models of inflammatory bowel disease and colon cancer, are needed to further confirm our hypothesis. The relatively simple chemical structure of boeravinones and the preliminary structure-antioxidant activity relationships presented here should be helpful in this task.

## Materials and Methods

### Chemicals

5,5-dimethyl-1-pyrroline-N-oxide (DMPO), hydrogen peroxide (H_2_O_2_), FeCl_2_·4H_2_O, trichloroacetic acid (TCA), thiobarbituric acid (TBA), malondialdehyde (MDA), 3-(4,5-dimethylthiazol-2-yl)-2,5-diphenyltetrazolium bromide (MTT), 2–7-dichlorofluorescein diacetate (DCFH-DA) and Phosphate buffered saline (PBS) tablets were purchased from Sigma-Aldrich (Milan, Italy). Monoclonal primary antibodies for pERK_1/2_, ERK_2_ and phospho-NF-kB p65 were obtained from Santa Cruz Laboratories (DBA S.r.l, Italy) while peroxidase-conjugated (HRP) anti-mouse IgG antibody was obtained from JacksonImmunoResearch (LiStarFish, Italy). All reagents for cell culture and western blot analysis were obtained from Sigma Aldrich S.r.l. (Milan, Italy), Amersham Biosciences Inc. (UK), Bio-Rad Laboratories (USA) and Microglass Heim S.r.l. (Naples, Italy). All chemicals and reagents employed in this study were of analytical grade.

### Plant material, extraction and isolation

Roots of *Boerhaavia diffusa* L. (Nyctaginaceae), collected in Bangalore (India), were kindly provided by Dr. Carlo Sessa, Milan. Fresh whole plants (2.20 lbs) including rootstocks and roots were extracted (3×3 L) with methanol at room temperature. Evaporation of the pooled extracts left a brown material (16.4 g) that was then subjected to a modified Kupchan's partition scheme [56] as follows. This crude methanol extract (BDME) was dissolved in MeOH-H_2_O 9∶1 and then partitioned against *n*-hexane (3×500 ml) to yield an apolar fraction weighing 1.55 g. Subsequently, the water content of the hydromethanolic phase was adjusted to 20% (v/v) and 40% (v/v) and the solutions partitioned against carbon tetrachloride (CCl_4_, 3×500 ml) and chloroform (CHCl_3_, 3×500 ml), respectively, affording CCl_4_ (g 0.56) and CHCl_3_ (g 0.90) fractions. Finally, all the MeOH was evaporated from the hydromethanolic layer, and the water solution thus obtained was partitioned against *n*-BuOH to yield butanol (1.32 g) and water (7.0 g) fractions. The CCl_4_ fraction was chromatographed by MPLC on silica gel (230–400 mesh) column (750×25 mm), using a linear gradient system (400 ml for each solvent) from *n*-hexane to EtOAc to MeOH-EtOAc (1∶1). The obtained fractions were pooled on the basis of their TLC behavior to afford 13 fractions called BOE-1 to BOE-13. Fractions BOE-4, BOE-6, BOE-8, and BOE-9, were further separated by HPLC. Both fractions BOE-4 (*n*-hexane-EtOAc, 8∶2) and BOE-6 (*n*-hexane-EtOAc, 7∶3) were purified by HPLC on an analytical column (250×4.6 mm) using *n*-hexane-EtOAc 75∶25 as eluent (flow rate 1.0 ml/min) and afforded as the main component boeravinone D (**1**, Figure 1, 6.4 mg). The BOE-8 fraction (eluted with hexane/EtOAc 6∶4) was purified by HPLC on an analytical column using hexane/EtOAc 7∶3 as eluent, flow rate 1.0 ml/min, obtaining boeravinone G (**2**, Figure 1, 7.5 mg). Finally, the BOE-9 fraction (eluted with hexane/EtOAc 1∶1) was purified by HPLC on an analytical column using hexane/EtOAc 6∶4 as eluent, flow rate 1.0 ml/min, obtaining boeravinone H (**3**, Figure 1, 5.2 mg). The chemical structures of these molecules were identified on the basis of the comparison of their spectral data with those reported in the literature [21], [22]. Boeravinone G was dissolved in dimethylsulphoxide (DMSO) to obtain a final concentration of 0.1% (w/w) in the culture medium; this drug vehicle had no effect on the responses under study.

### Cell culture

Human colon adenocarcinoma Caco-2 cells were purchased from the American Type Culture Collection (LGC Promochen, Italy) and used between passages 30 to 50. The cells were routinely maintained in 75 cm^2^ polystyrene flasks in growth media consisting of DMEM (Dulbecco's Modified Eagle Medium) containing 10% Fetal Bovine Serum (FBS), 100 U/ml penicillin, 100 µg/ml streptomycin, 1 M Hepes [4-(2-Hydroxyethyl)-1-piperazineethanesulfonic acid] 2.5%, non-essential amino acid (NEAA) 1×, 2 mM L-glutamine at 37°C in a 5% CO_2_ atmosphere. The medium was changed every 48 h. For cell vitality, lactate dehydrogenase leakage, TBARS and ROS assays cells were led to differentiation, (cells were used at post-confluence stage as a model of human enterocytes); preliminary experiments showed that a 7-day time of incubation was required for Caco-2 cells to undergo differentiation.

### Hydroxyl radical generation and detection (electron spin resonance spectroscopy)

Hydroxyl free radicals were obtained by the Fenton reaction: 0.2 ml H_2_O_2_ (10 mM) and 0.2 ml FeCl_2_·4H_2_O (10 mM) mixed with 0.2 ml 5,5-dimethyl-1-pyrroline-N-oxide (DMPO, 0.3 M) used as spin trap (blank). Hydroxyl radicals production was detected by the ESR spectrometer Bruker 3000E (Rheinstetten, Germany) with the following settings: field modulation 100 kHz, modulation amplitude 0.512 G, receiver gain 2×10^5^, time constant 81.92 ms, conversion time 163.84 ms, center field 3440.00 G, sweep width 100.00 G, x-band frequency 9.64 GHz, power 20 mW, temperature 23°C [57]. The influence of BDME and its fractions/constituents on the formation and transformation of hydroxyl radicals was investigated by adding the extract to the Fenton reaction system in the range of concentrations 0.10–5 mg/ml and 0.01–1 ml for BDME and fractions, respectively. The ESR spectra were recorded after 5 min. The antioxidant activity (AA) value of the extract was defined as: AA = 100×(h_0_−h_x_)/h_o_ (%) where h_o_ and h_x_ are the height of the first peak in the ESR spectrum of DMPO-OH spin adduct of the blank and the probe, respectively.

### TBARS assay

Lipid peroxidation products [thiobarbituric acid reactive substances (TBARS) also known as malondialdehyde-equivalents (MDA-equivalents)] from Caco-2 cells were measured by the thiobarbituric acid colorimetric assay. Briefly, Caco-2 cells were seeded in 6-well plates at the density of 3.0×10^6^ and led to differentiation. Differentiated cells were treated with boeravinone G (0.1–1 ng/ml corresponding to 0.28–2.8 nM) for 24 h and then washed with PBS and incubated with the Fenton's reagent (H_2_O_2_/Fe^2+^ 1 mM) for 3 h at 37°C. The 1 mM concentration was selected on the basis of our preliminary experiments, which showed submaximal effects of H_2_O_2_/Fe^2+^ in this assay (pmol MDA/mg protein: control 182.1±17.90, H_2_O_2_/Fe^2+^ 0.25 mM 182.6±20.68, H_2_O_2_/Fe^2+^ 0.5 mM 298.5±17.62, H_2_O_2_/Fe^2+^ 1 mM 628.5±34.58, H_2_O_2_/Fe^2+^ 2 mM 985.5±62.5, H_2_O_2_/Fe^2+^ 4 mM 1039±62.3; n = 8. EC_50_: 0.96±0.07 mM, E_max_: 1052±50.37%). After incubation, the cells were washed and scraped in ice cold PBS. The cells were lysed by six cycles of freezing and thawing in PBS and then centrifuged at 16200× g for 10 min at 4°C. Trichloroacetic acid (TCA, 10% w/v) was added to the cellular lysate and, after centrifugation at 16200× g for 10 min, 0.67% (w/v) thiobarbituric acid (TBA) was added to the supernatant and the mixture was heated at 80°C for 30 min. After cooling, MDA-equivalents formation was recorded at the wavelength of 532 nm, using a Beckman DU62 spectrophotometer. A standard curve of MDA was used to quantify the levels of MDA-equivalents formed during the experiments, and the results are presented as µmol of MDA-equivalents/mg of cell protein previously determined by the Bradford method [58].

### Detection of reactive oxygen species (ROS) generation

Generation of intracellular reactive oxygen species (ROS) was estimated by a fluorescent probe, DCFH-DA [59]. DCFH-DA diffuses readily through the cell membrane and is enzymatically hydrolyzed by intracellular esterases to form non-fluorescent DCFH, which is then rapidly oxidized to form highly fluorescent DCF in the presence of ROS. The DCF fluorescence intensity is paralleled to the amount of ROS formed intracellularly. For experiments, cells were plated in a 96 multiwell plate at the density of 1×10^4^ cells/well and led to differentiation. Confluent Caco-2 cell monolayers were incubated for 24 h at 37°C with boerhavinone G (0.1–1 ng/ml). Then, the cells were rinsed and incubated for 30 min with 100 µM DCFH-DA in Hanks' Balanced Salt Solution (HBSS) containing 1% FBS. Finally, cells were rinsed and incubated with the Fenton's reagent (H_2_O_2_/Fe^2+^ 2 mM) for 3 h at 37°C. The 2 mM concentration of H_2_O_2_/Fe^2+^ induced a submaximal increase in ROS production (control 2.26±0.19, H_2_O_2_/Fe^2+^ 0.5 mM 2.61±0.19, H_2_O_2_/Fe^2+^ 1 mM 10.29±1.27, H_2_O_2_/Fe^2+^ 2 mM 27.34±1.29, H_2_O_2_/Fe^2+^ 3 mM 39.43±3.13, H_2_O_2_/Fe^2+^ 4 mM 44.23±2.31; n = 8. EC_50_: 2.29±0.07 mM, E_max_: 54.40±9.11%). The DCF fluorescence intensity was detected using a fluorescent microplate reader (Perkin-Elmer Instruments), with the excitation wavelength of 485 nm and the emission wavelength of 538 nm.

### DNA damage assay

The presence of DNA fragmentation was examined by single cell gel electrophoresis (Comet assay), as previously described [60]. Briefly, Caco-2 cells were seeded in the 25 cm^2^ flasks at a density of 4×10^5^ cells and incubated with boeravinone G (0.1–1 ng/ml) at 37°C for 24 h. After incubation the cells were treated with H_2_O_2_ (75 µM) for 5 min on ice and then centrifuged at 1000× g for 5 min. This concentration of H_2_O_2_ produced a submaximal damage of DNA (data not shown). The supernatant was discarded and the pellet was mixed with 85 µl of 0.85% low melting point agarose (LMA) in PBS. Cells were added to previously prepared gels of 1% normal agarose (NMA). The gels on frosted slides were maintained in lysis solution (2.5 M NaCl, 100 mM Na_2_EDTA, 10 mM Tris and 1% Triton X-100, pH 10) at 4°C for 1 h, and then electrophoresed in an appropriate buffer (300 mM NaOH, 1 mM Na_2_EDTA, pH>12) at 26 V, 300 mA for 20 min. After running, the gels were neutralized in 0.4 M Tris–HCl, pH 7.5 (3×5 min washes) and stained with 20 µl of ethidium bromide (2 µg/ml) before scoring. Images were analyzed using a fluorescence microscope (Nikon) interfaced with a computer. DNA damage was analyzed and quantified by measuring the percent of fluorescence intensity in the tail (tail intensity) through the Komet 5.0 image analysis software (Kinetic Imaging). Each treatment was carried out in duplicate, and 100 random selected comets from two microscope slides were analyzed.

### Preparation of cytosolic fractions

Caco-2 cytosolic extracts were prepared as previously described [60]. Briefly, after boeravinone G (0.1–1 ng/ml) incubation for 24 hours followed by a treatment with H_2_O_2_/Fe^2+^ 1 mM for 3 hours, the medium was removed and cells were washed with ice cold PBS. The cells were collected by scraping for 10 min at 4°C with lysis buffer [50 mM Tris-HCl pH = 7.4, 0.25% sodium deoxycholate, 150 mM NaCl, 1 mM EGTA, 1 mM NaF, 1% NP-40, 1 mM PMSF, 1 mM Na_3_VO_4_ containing complete protease inhibitor cocktail (Roche Diagnostics, Mannheim, Germany)]. After centrifugation at 16,200× g for 15 min at 4°C, the supernatants were collected and protein concentration was determined by Bradford method [58]. Cytosolic lysates were used for the evaluation of SOD activity, and pERK_1/2_ and phospho-NF-kB p65 levels.

### Superoxide dismutase (SOD) assay

A modified version of the Kuthan and colleagues method was used to detect SOD activity [61]. In this method, superoxide radicals were generated using a xanthine oxidase/hypoxanthine system, and the ability of cells to scavenge superoxide radicals was measured spectrophotometrically. Cytosolic lysates were incubated at 25°C for 20 min with a reaction mixture containing 1.2 mM xanthine, 0.03 mM nitro blue tetrazolium (NBT), 0.26 U/mL xanthine oxidase. Absorbance readings at 560 nm were recorded using a Beckman DU62 spectrophotometer. Superoxide radical-scavenging capacity of boeravinone G (0.1–1 ng/ml) at the end of 30 min were expressed as ng SOD/mg proteins contained in the cell lysates.

### Western blot analysis

Proteins (50 µg) were subjected to electrophoresis on an SDS 12% polyacrylamide gel and electrophoretically transferred onto a nitrocellulose transfer membrane (Protran, Schleicher & Schuell, Germany). The immunoblots were developed with 1∶1000 dilution for pERK_1_, pERK_2_ and phospho-NF-kB p65, and the signals were detected with the ECL System according to the manufacturer's instructions (Amersham Pharmacia Biotech). The membranes were probed with an anti-ERK_2_ and anti-βactin antibody, to normalize the results, which were expressed as a ratio of densitometric analysis of pERK_1/2_/ERK_2_ and phospho-NF-kB p65/β-actin bands, respectively.

### Cell viability

Cellular viability was assessed by the MTT [3-(4,5-dimethylthiazol-2-yl)-2,5-diphenyltetrazolium bromide] assay as described by Mosmann [62]. Caco-2 cells were plated in a 96 multiwell plate at a density of 1×10^4^ cells/well. After differentiation the cells were treated with vehicle (DMSO 0.1% v/v) or boeravinone G (0.1–1 ng/ml) for 24 h and, then, incubated with the MTT solution (0.25 mg/ml) for 1 h at 37°C. The supernatant was removed and the formed formazan crystals were dissolved in DMSO (100 µl/well) at room temperature for 10 min. The absorbance was read at the wavelength of 490 nm in a multiwell plate reader (Bio-Rad, Model 550). The mean absorbance, taken from cells grown in the absence of the extracts (vehicle alone), was taken as 100% cell survival (control).

### Lactate dehydrogenase (LDH) leakage assay

The injury to Caco-2 cells was quantitatively assessed through the measurement of lactate dehydrogenase (LDH) levels. Caco-2 cells were seeded in 6-well plates at the density of 3.0×10^6^ and led to differentiation. Differentiated cells were treated with vehicle (DMSO 0.1% v/v) or boeravinone G (0.1–1 ng/ml) for 24 h. An aliquot of the medium was removed from the culture plates and then analyzed for LDH leakage into the culture media by using a commercial kit (Sigma Diagnostics). The total LDH activity was determined after cells were scraped and thoroughly disrupted by Ultra Turax for 30 seconds. The percentage of LDH leakage was calculated to determine membrane integrity. The LDH leakage was expressed as a percentage of the total activity: (activity in the medium)/(activity in the medium+activity of the cells)×100.

### Statistical analysis

Statistical analysis was carried out using GraphPad Prism 4.01 (GraphPad Software, San Diego, CA, USA). All data were expressed as mean ± SEM of duplicate determinations and are representative of at least three determinations. Groups of data were compared with the analysis of variance (ANOVA) followed by Tukey's multiple comparison tests. Values of *P*<0.05 were regarded as significant. The IC_50_ and E_max_ values were calculated by nonlinear regression analysis using the equation for a sigmoid concentration– response curve (GraphPad Prism).
